# Editorial: Pathophysiological and clinical advances in asthmatic inflammation from the nasopharynx to the peripheral airway in the respiratory tract systems

**DOI:** 10.3389/fphar.2023.1037610

**Published:** 2023-01-16

**Authors:** Yasuhiko Koga, Yosuke Kamide, Takeshi Hisada, Tamotsu Ishizuka

**Affiliations:** ^1^ Department of Respiratory Medicine, Gunma University Graduate School of Medicine, Maebashi, Gunma, Japan; ^2^ Clinical Research Center for Allergy and Rheumatology, Sagamihara National Hospital, Sagamihara, Japan; ^3^ Gunma University Graduate School of Health Sciences, Maebashi, Gunma, Japan; ^4^ Third Department of Internal Medicine, Faculty of Medical Sciences, University of Fukui, Fukui, Japan

**Keywords:** metformin, leptin, eosinophilic chronic rhinosinusitis, severe asthma, omalizumab, type IV collagen, benralizumab, non-coding RNA

Asthmatic airway inflammation is associated with many diseases, ranging from the upper to the lower respiratory tract, such as eosinophilic chronic sinusitis with nasal polyps (ECRSwNP), childhood and adult asthma, eosinophilic bronchiolitis/pneumonia, and eosinophilic granulomatosis with polyangiitis (EGPA). Various biological agents have been introduced for patients refractory to oral corticosteroid-based therapy. However, the mechanism of asthma-related inflammation remains obscure, and overcoming this intractable disease remains challenging. In this Research Topic, we focused on various asthmatic inflammatory diseases to deepen the understanding of underlying complicated pathological mechanisms, and the efficacy of the latest biologics against asthmatic inflammation.

**FIGURE 1:**
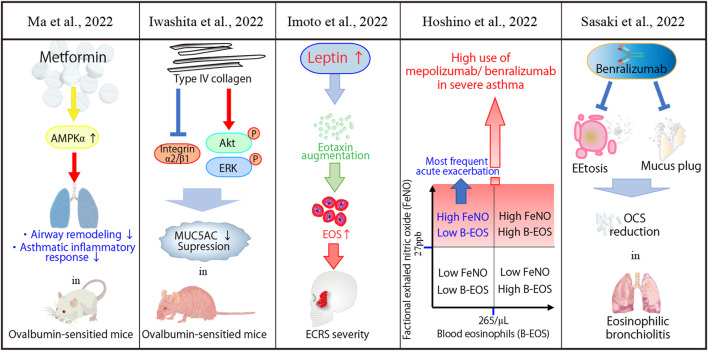
A brief summary of articles and a case report are shown.

An *in vivo* study has demonstrated the beneficial effects of classical hypoglycemic agent, metformin, in metabolic syndrome, cancer, and chronic inflammation and tissue-remodeling through AMPK-dependent or AMPK-independent mechanisms (Saisho, 2015; Wu et al., 2021). Furthermore, metformin alleviates airway inflammation in asthmatic patients with obesity (Guo et al., 2021). As shown in Figure 1, Ma et al. clarified the mechanisms of metformin-mediated improvement in the airway inflammatory cell infiltration by restoring AMPKα activity using ovalbumin-sensitized asthmatic mice. Articles and reviews on this Research Topic are summarized in Figure 1; Table 1, respectively. Moreover, Iwashita et al. demonstrated that type IV collagen, an extracellular matrix protein, suppresses MUC5AC secretion by regulating integrin α2 and β1 expression in the lungs and increases Akt and ERK phosphorylation using ovalbumin-sensitized asthmatic mice. This study was consistent with a previous *in vitro* study (Iwashita et al., 2010). These *in vivo* studies indicate that alternative treatment options may be promising for refractory asthmatic inflammatory diseases.

**TABLE 1:** Summary of reviews.

Review theme	Main Topics	Future Prospectives
Non-coding RNAs in childhood asthma Liang et al. (2022)	・Long non-coding RNAs regulates Th1/Th2 imbalance, Treg/Th17 imbalance, eosinophils dysfunction, ASMCs proliferation	・Non-coding RNAs are promissing for diagnositic biomarker and threrapeutic targets in childhood asthma
	・Micro RNAs regulate Th1↓/Th2↑ balance, ASMCs proliferation, and inhibit IL-13 secretion	
	・Circular RNAs regulate the secretion IL-13/IL-6 and promote ASMCs proliferation	
	・Non-coding RNAs involved in airway remodeling	
Effect of benralizumab in EGPA Koga et al. (2022)	・Summary of 41 patients with EGPA treated with benralizumab	・Promissing effect for mepolizumab-refractory EGPA
	・Benralizumab depletes both cirlular and tissue eosinophils *via* ADCC activity	・Promissing effect cardiac and nerve involvements of EGPA
	・Discontinuation of OCSs was achieved in more than 40% patients with EGPA	・RCT has been evaluating the efficacy and safety of benralizumab (30 mg) compared to mepolizumab (300 mg)
Significant monitoring free IgE in patients with asthma treated with omalizumab Gon et al. (2022)	・Half life elongation of free IgE plus omalizumab complexes (free IgE *versus* IgE + omalizumab: 2.4 days *versus* 20 days)	・During omalizumab therapy, revisable dosage of omalizumab by free IgE measurement using FcεRIα recombinant protein as a clinical option
	・Omalizumab-IgE complexes promote lower free IgE production by suppressing the binding of CD23 and IgE	
	・Monitoring of free IgE in severe asthma could help predict omalizumab sensitivity	

Th, helper T cell; Treg, regulatory T cell; ASMCs, airway smooth muscle cells; EGPA, eosinophilic granulomatosis with polyangiitis ADCC, antibody-dependent cell-mediated cytotoxicity; OCSs, oral corticosteroids; RCT, randomized clinical study.

Imoto et al. revealed an association between serum leptin levels, a hormone secreted by adipocytes (Bado et al., 1998), and eosinophilic chronic sinusitis (ECRS), a subtype of CRS with nasal polyps. Serum leptin levels have been associated with eosinophilia and eosinophilic infiltration of polyp tissues in patients with CRSwNP. Additionally, it has been correlated with ECRS severity. Interestingly, leptin significantly augmented eotaxin-3 expression, *in vitro*, in human primary cultured nasal fibroblasts, showing the correlation between serum leptin levels and eotaxin-3 mRNA expression in nasal polyps.

Liang et al. reviewed the association between non-coding RNA (ncRNA) and childhood asthma; ncRNA (does not encode proteins) mainly including microRNAs (miRNAs), long non-coding RNAs (lncRNAs), and circular RNAs (circRNAs) (Huang and Xiong, 2020). Both lncRNAs and miRNAs are crucial for pathogenesis and abnormal regulation of childhood asthma. Further, lncRNAs are associated with Th2-related cytokines (IL-5 and IL-13) and transcription factors, and chemokines affect the balance of Th1/Th2, thus causing asthma (Wang et al., 2017). Reports have shown that miRNAs may participate in the pathogenesis of childhood asthma by increasing Th2 cytokine secretion, decreasing Th1 cytokine secretion, and promoting the differentiation of CD4^+^ T cells into Th2, thereby causing airway inflammation (Midyat et al., 2016). Thus, the discovery of lncRNAs and miRNAs has furthered our understanding of childhood asthma (Rundell et al., 2015); ncRNAs are considered potential biomarkers and promising therapeutic targets for childhood asthma (Narozna et al., 2017; Specjalski and Jassem, 2019).

One clinical retrospective study, one case report, and two reviews reported the clinical significance of biologics for airway diseases featuring asthmatic inflammation. Hoshino et al. investigated the phenotype of severe asthma in predicting sputum eosinophilia. A total of 114 adult patients with severe asthma were stratified into four subgroups defined by the thresholds of the fraction of exhaled nitric oxide (FeNO) and blood eosinophil (B-EOS) counts predicting sputum eosinophilia. The sputum eosinophil-predominant subtype was highest in the high FeNO/high B-EOS. The high FeNO/high B-EOS and high FeNO/low B-EOS subgroups had the highest prevalence of mepolizumab and benralizumab use, respectively. The high FeNO/low B-EOS exhibited the largest frequency of acute exacerbation (AE) compared with the other FeNO/B-EOS groups. This study suggested that classification based on the combination of FeNO and B-EOS proposes a specific refractory type 2 severe asthma, thus causing optimal biologics use. Sasaki et al. reported a case of EEtosis in the mucus plugs of a patient with eosinophilic bronchiolitis, successfully treated with benralizumab. A recent population-based cohort study in Taiwan revealed that patients with a recent (<3 years) and older age (> 30 years of age) diagnosis of asthma had a higher probability of developing hyperthyroidism (Gau et al., 2022). While asthma is caused by T helper 2, hyperthyroidism is thought to be caused by T helper 1. We need to recognize further that adults with asthma are at higher risk of developing hyperthyroidism.

Benralizumab, an anti-interleukin-5 receptor α antibody, successfully stabilized the patient’s condition and reduced systemic corticosteroids. Here, we reviewed the safety and efficacy of benralizumab as a promising treatment option for refractory EGPA. In total 41 patients with EGPA treated with benralizumab were reviewed. After administrating benralizumab, oral corticosteroids were reduced to ≤ 10 mg/day in all cases and ≤ 5 mg/day in 80% or more cases, and their discontinuation was achieved in > 40% of cases. Benralizumab was effective in patients with mepolizumab-refractory EGPA and intractable cardiac and neuropathy complications (Colantuono et al., 2020; Nanzer et al., 2020; Padoan et al., 2020; Bormioli et al., 2021). Recently, long-term safety and efficacy of benralizumab for > 4 years were reported in a case of EGPA complicated by severe neuropathy (Koga et al., 2022). Two ongoing clinical trials are evaluating the safety and efficacy of benralizumab in patients with EGPA treated with OCS; (BITE) (NCT03010436) and (MANDARA) (NCT04157348).

Gon et al. reviewed the relationship between IgE-targeted therapy and serum IgE levels to enhance the current understanding of the mechanism of omalizumab therapy. Total serum IgE levels increased after omalizumab therapy compared to pre-administration levels due to the differences in half-time between serum IgE (2.4 days) and serum IgE plus omalizumab complexes (20 days). Methods for measuring free IgE levels in the presence of omalizumab antibodies have been identified (Baker et al., 2014). A prospective study revealed the significance of measuring serum free IgE level using the IgE measurement method and FcεRIα recombinant protein with the human glycosylation structure (Ito et al., 2014). Measurement of serum free IgE levels during omalizumab therapy is recommended to revise the dosage of omalizumab. Besides using serum free IgE for predicting the therapeutic effects of omalizumab, quantifying serum free IgE has been suggested to be beneficial (Tajiri et al., 2016). Dupilumab, antagonizing an anti-IL-4 receptor α together with IL-13 receptor, includes indications for phenotypes of severe asthma that are remarkably overlapping with indications of omalizumab (Salvati et al., 2022). These biologics showed excellent benefits in patients with severe asthma complicating other Th2 disorders such as nasal polyps, seasonal allergic rhinitis, chronic spontaneous urticaria, CRSwNP, and atopic dermatitis.

Metformin, type IV collagen, and leptin are negatively and positively associated with asthmatic inflammation. Furthermore, benralizumab is a promising agent for asthmatic refractory airway diseases. Monitoring serum free IgE levels during omalizumab treatment may be utilized with further investigation.
